# The COVID-19 Pandemic: Public Health and Epidemiology

**DOI:** 10.1177/1010539520929223

**Published:** 2020-05-19

**Authors:** Colin Binns, Wah Yun Low, Lee Mi Kyung

**Affiliations:** 1 School of Public Health, Curtin University, Perth, Western Australia, Australia; 2 Faculty of Medicine, University of Malaya, Kuala Lumpur, Malaysia; 3 Asia-Europe Institute, University of Malaya, Kuala Lumpur, Malaysia; 4 College of Science, Health, Engineering and Education, Murdoch University, Perth, Western Australia, Australia

In this issue of the journal, we publish a review of COVID-19 infection by 2 eminent virologists, MacKenzie and Smith (see in this issue). It is too early in the history of the COVID-19 outbreak to write the full history, but their article provides a good outline of the emerging pandemic. The disease is causing widespread social disruption in many countries, and it has just been announced that the 2020 Asia Pacific Academic Consortium for Public Health (APACPH) Conference has been postponed indefinitely. The media are full of daily totals of new cases, hospital and intensive care admissions, and deaths. The actual numbers are dependent on the testing regimes in use at different locations. In the first 2 months of 2020, there were hundreds of papers and commentaries published on Corona viruses, including a major clinical review in the *Lancet* that has already had almost 500 citations.^1^ Corona viruses are a large family of viruses that can cause human diseases, but usually mild in nature, such as a common cold. There have been two previous severe outbreaks of novel corona viruses: severe acute respiratory syndrome (SARS) in 2003 and Middle East respiratory syndrome coronavirus (MERS-CoV) in 2012, which together caused more than 10 000 cases. The case fatality rates (CFRs) were 10% for SARS-CoV and 37% for MERS-CoV.^1^ In this commentary, we will discuss additional public health issues that will assist in teaching at our public health institutions.

Since the first cases of COVID-19, the reported numbers have increased rapidly with more than 1.2 million cases and 3200 deaths to the end of February 2020, and cases are now being reported from all of the more populous countries.^2^ The symptoms of COVID-19 infection are nonspecific and include elevated temperature and cough. This, then, progresses to shortness of breath.^3^ A clinical report of the first patients from Wuhan (n = 41) with COVID-19 infection gave the following details of clinical symptoms: fever 98%, cough 76%, myalgia or fatigue 44%, sputum 28%, and dyspnea 55%. The average time from the first onset of symptoms to the development of dyspnea was 8 days. In the first report from outside of Wuhan (from Zhejiang Province, n = 62), the symptoms were fever 77%, cough 81%, sputum 56%, headache 34%, and myalgia or fatigue 52%. The disease is more likely to occur in people who have a chronic illness, are immunocompromised, or are older. Those who contracted the virus and were older than 60 years of age, and particularly older than 80 years, were more likely to require admission to intensive care unit, and have an increased CFR^4^ (see Table 1 in Mackenzie and Smith, this issue). In Wuhan, there were 1023 deaths from the 44 672 confirmed cases, an overall CFR of 2.3%.

The initial clinical symptoms of COVID-19 are the symptoms of a common cold and influenza. Every adult gets 2 to 3 colds per year, and more frequently in children.^5^ With a world population of around 7.8 billion, this suggests that there are 20 billion cases every year. Screening all of these cases for possible COVID-19 would obviously be impossible, and so testing for the virus is confined to those with other risk factors, including contact with confirmed cases and travel from outbreak epicenters.

COVID-19 is the latest in a continuing series of infectious disease epidemics in the history of the human race. As population numbers and population density increases, the likelihood of epidemics increases. Probably, the greatest epidemic of all time was the influenza epidemic of 1918, which caused an estimated 50 million deaths worldwide and had a CFR of more than 2.5% and which could have been as high as 10%.^6^ This was in the pre-antibiotic days, and before an influenza vaccine and antiviral therapy had been developed. Infectious diseases remain a major challenge for public health.

To assist in defining, containing, preventing, and ultimately in successfully treating those who become ill in an epidemic, it is important to understand the basic epidemiology of the outbreak. Experts from many institutions have collaborated together in an effort to categorize the COVID-19 outbreak. Most infectious diseases have a spectrum of severity from clinically undetected disease through to death.^7^ Figure 1 is modified from the paper prepared by Imperial College. It is likely that only the cases with severe symptoms or other risk factors (e.g. contact with a known case) are being identified at present, but it varies between countries.

**Figure 1.**
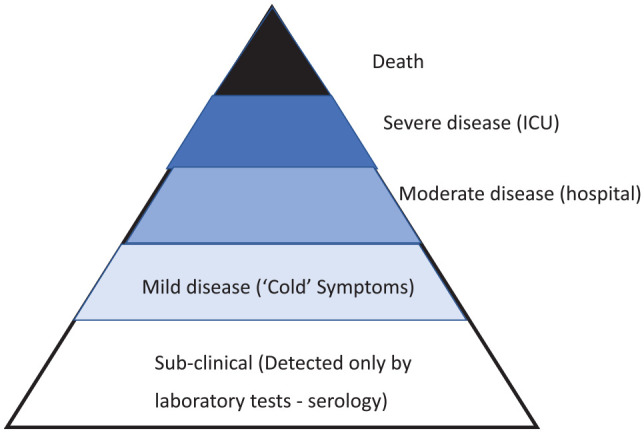
The clinical spectrum of COVID-19 infection.

For some diseases, the base of the triangle in Figure 1 would be much wider, and this may be the situation with COVID-19. The base of subclinical cases with minimal symptoms may be much greater than those with disease severe enough for hospital admission. The history of public health contains a number of examples of infectious diseases that were initially thought to have had a very high CFR only for it to be revised downward later. Lassa fever was initially described as a very severe disease, with a CFR close to 100%. However, as more widespread epidemiological studies were undertaken in West Africa, it was found that 50% or more of the population of Sierra Leone and Guinea were seropositive to the disease without showing any symptoms.^8^ Under these circumstances, the CFR for Lassa virus is now thought to be low (<1%), but the CFR for severe disease remains very high.

## Definitions (Some Terms in the Epidemiology)

When assessing the likely impact of an infectious disease, there are two parameters that are considered: the likelihood of transmission of the disease (its capacity to spread), and the severity of the disease and its capacity to kill (or disable) those infected. These are assessed using the reproduction rate and the CFR.

*Reproduction rate* (*R*_0_): The base reproduction number (*R*_0_) for transmission indicates the number of secondary infections due to an initial case (*R*_0_ = virgin state, a population with no previous exposure to this infection^9^). In populations that have previously been exposed to the infection, and have some immunity, the reproduction rate may be lower.

Case fatality rate is defined as the proportion of reported cases of a specified disease that are fatal within a specified time.^10^ The CFR depends on the definition of disease, the accuracy of diagnosis (case detection), and the availability of treatment.

Transmissibility and severity are the two most critical factors that determine the public health impact of an epidemic.^11^ A disease that has a high transmission rate and is very severe is the greatest public health risk. COVID-19 has a high transmission rate, and the CFR appears to be greater than for influenza epidemics, and it is, therefore, potentially a major public health threat.

Infectivity is the tendency to spread the infection from host to host. The period of infectivity for some diseases commences before symptoms appear, making it far more difficult to control spread.

The spectrum of clinical disease (see Figure 1) makes it difficult to define a case and even more difficult to calculate a CFR, as the denominator is not readily defined. In February, the CFR for COVID-19 infection was estimated by the World Health Organization (WHO) to be 2%, much lower than for MERS and SARS, but estimates of the CFR have changed over time as the criteria for counting the number of cases in the denominator has changed to include very mild or even asymptomatic infections.^12^ Changes in the denominator decrease the CFR and increase the reproducibility rate. In some situations, the reproducibility rate changes and the quarantine of passengers on a cruise ship in Yokohama had a *R*_0_ 4 times greater (as high as 14) compared with the initial *R*_0_ in Wuhan.^9^

The use of diagnostic tests has traditionally been reported using measures of sensitivity (proportion of true positives that are correctly identified by the test) and specificity (proportion of true negatives that are correctly identified by the test).^13^ At the present time, COVID-19 disease is diagnosed by detecting the virus in throat and nasal swabs in patients who have symptoms of an upper respiratory tract infection who have been in a region with disease transmission. No other screening tests are yet available, and in the absence of “gold standard” for diagnosis, these parameters are not yet being calculated.

Incubation period is the time between exposure to an infectious agent and the appearance of clinical symptoms (or physiological evidence of disease). It is not known if transmission of the virus occurs during this period and before the presence of clinical symptoms. The WHO estimates of the incubation period for COVID-19 range from 1 to 14 days, most commonly around 5 days. Modeling of the role of contact tracing and case isolation suggest that these are effective in the control of epidemics such as COVID-19.^14^ In Australia, the home isolation (quarantine) of contacts or suspected cases is recommended for 14 days.^15^ If transmission is occurring before symptoms appear, it makes it more difficult to control an infectious disease.

The virus is contained in droplets from coughing and nasal secretions. It can survive up to 24 hours on surfaces under favorable conditions. Most transmission will occur through spread by hands and not from the direct inhalation of droplets. The WHO recommends the following to prevent transmission of the virus:

- Regularly and thoroughly clean your hands with an alcohol-based hand rub or wash them with soap and water.
- Maintain at least 1 m distance between yourself and others, and in particular from anyone who is coughing or sneezing.
- Avoid touching eyes, nose, and mouth.
- Make sure you, and the people around you, follow good respiratory hygiene. This means covering your mouth and nose with your bent elbow or tissue when you cough or sneeze. Then dispose of the used tissue immediately.
- Stay at home if you feel unwell. If you have a fever, cough, and difficulty breathing, seek medical attention and call in advance. Follow the directions of your local health authority.^16^

## Vaccine Development

Despite much research and success in some animal models, including primates, there is still no vaccine for SARS. The first clinical cases of SARS were noted in November 2002, but it was not until 5 months later that a causative agent was isolated.^17^ Transmission and dispersion around the world occurred by respiratory aerosols and contact via hands, and in several cases, this was from touching door handles. There were 8098 reported cases of SARS and 774 deaths from many different countries.^18^ While there is no doubt that the virus has the potential to reemerge at present, it is not a clinical issue at present. SARS has a lower transmissibility than COVID-19.

The development, testing, and mass production of vaccines is always time-consuming before they can be deployed on a population-wide scale. The development, testing, and distribution of a vaccine will take years. Despite the interest in SARS, there is still no vaccine, and in other cases, important diseases have defied vaccine development, notably malaria and dengue.

## Seasonality

Will COVID-19 decline in the northern summer following the pattern of influenza? This may be because people are more likely to have closer contact with others because it is colder. It could also relate to relative humidity levels that are lower in winter. In Western countries, when it is cooler in winter, respiratory infections increase (e.g. influenza). However, this seasonality does not apply to influenza in India.^19^ A study of a new strain of influenza in Vietnam shows that in the early years of this variant, there was significant transmission throughout the first year and the usual seasonal transmission pattern evolved a few years later.^20^ In Australia, it is summer and hot, and the COVID-19 is spreading here. This suggests that COVID-19 transmission may not be related to climatic conditions and may not be seasonal in its early year(s) as the population is being exposed for the first time.

## Smallpox as an Example of the Role of Epidemiology in Disease Control

The history of smallpox elimination provides an example of the role of epidemiology in defeating an infectious disease. The practice of variolation to prevent smallpox had been in existence for several centuries, particularly in the Middle East, before Edward Jenner popularized the process and published his paper on the topic.^21^ Vaccination against smallpox developed for almost 2 centuries before the disease was certified as being eliminated in 1980.^22^ What was needed was a thorough understanding of the epidemiology of the disease. By studying seasonal variation and transmission within an infection cluster, it was shown that with vaccination of perhaps 5% of the population, the immediate case contacts was just as effective as vaccinating 100% of the population.^23^ Understanding the epidemiology of outbreaks proved to be the effective way of eliminating the disease.^24^ Recording all of the details of the COVID-19 outbreak is basic to understanding its epidemiology and the natural history of the disease and could provide the key to defeating the disease outbreak.

## Health Ethics

There are several ethical issues that have already been raised in this outbreak. The importance of preserving the physical and mental health and availability of health workers in an epidemic situation is very important. The quality of health care depends almost entirely on them having a professional service ethic that motivates them to provide the highest quality care to all with the resources available to them.^25^ This often leads them to put their own health and safety at risk, especially in infectious disease outbreaks. After the SARS outbreak of 2003 that caused illness and deaths among health workers, the ethics of exposing staff to the disease was still being debated in 2019.^26^ The history of public health is full of the writings of the heroism of health workers providing care to those in need, despite endangering themselves.^27^ However, experience has shown that risks associated with outbreaks of life-threatening infections only receive attention after health workers have suffered serious adverse consequences. Institutions need to prepare for outbreaks and provide the best available protective equipment to their workers and volunteers.

A further ethical issue is the development of vaccines. If this proves possible, it will take several years to develop, test, and submit for approval. Then issues of distribution and cost will need to be finalized. Professor Jeffrey Sachs, health economist, public health advocate, and former advisor to the Secretary General of the United Nations for the development of the Sustainable Development Goals, has written an editorial on the development of a vaccine for COVID-19.^28^ Sachs states that in earlier public health emergencies, governments, nonprofit foundations, and international organizations took the lead in the development of preventive measures and made the vaccine or knowledge available freely. Sachs discusses the example of Jonas Salk who did not patent the polio vaccine, making it affordable to programs all over the world. However, in the case of COVID-19, the present US administration is stating that commercial companies will develop the vaccine and its availability will depend on the market. Will the profits of multinational companies come before the survival of the poor in lower income countries^28^?

## Public Health Actions

Reducing the peak of new cases by slowing down the spread of new cases is very useful as it

1. Reduces the load on diagnostic and treatment services
2. Reduces the number of health workers who contact the disease and who can continue working
3. If the epidemic curve can be smoothed, it will result in a lower overall disease burden

There are examples from the historical control of epidemics using public health measures that may be applied to COVID-19. An analysis of records from 17 cities in the United States during the 1918 influenza pandemic shows that cities that implemented public health measures (isolation, banning meetings, etc) were successful in reducing epidemic peaks and overall mortality.^29^ This was in a naive population not previously exposed to this virus. Hatchett et al documented the substantial differences between St. Louis (which banned mass gatherings) and the higher death rates in Philadelphia, which allowed the massed celebratory marches following World War I to proceed.^29^

Useful public health interventions to reduce the peak of new cases include the following:

1. *Personal hygiene*: no handshaking, no direct physical contact with others, keeping 1 m distance from others, no coughing in public, do not touch your face, wash hands frequently with soap and water, and eat cooked food that is still hot. These measures require clean water supplies (also important for nutrition and food safety)
2. Reducing person to person contact by banning public gatherings, closing schools, home quarantining, controlling public transport, and so on, has been effective, but disruptive, in some communities
3. Isolation of cases and quarantine (usually self-quarantine) of contacts.

There is more information on the WHO and Centers for Disease Control and Prevention websites.^2,3^

In their modeling, Hellewell et al suggest that isolation of cases and tracing of contacts may be successful in controlling an outbreak within 3 months.^14^ In the current global situation, it appears that some countries have been able to slow the epidemic while others have been overwhelmed by peaks of cases.

Universities are presented with major challenges to manage the COVID-19 epidemic. They are gathering places for thousands of young adults and academics who are usually older and are more vulnerable to complications. Obviously, large gatherings should be avoided and wider use be made of online teaching and tutorials. On campus, the usual precautions of avoiding contact, keeping distance, and frequent handwashing should apply. Within the memory of one of us (CB), these personal precautions were applied with success to polio, influenza, and hepatitis. We hope that these precautions will lead to a containment of the epidemic allowing time to continue development and clinical trials of a vaccine.

## Conclusion

The emergence of COVID-19 is a serious global public health problem. The future direction of the epidemic is unknown. The size of the outbreak will depend on reducing transmission, which at the present time means using traditional public health measures. These include contact tracing and quarantine of cases, or sometimes the quarantine of localities. Modeling suggests that these measures may be effective. Health promotion programs should emphasize avoiding crowds, handwashing and hygiene, and extensive testing of at-risk persons. Vaccine development is a slow process, and it will be a year(s) before it can become a component of public health interventions.

Schools of Public Health and Research Institute members of APACPH are actively involved in basic research, epidemiology of outbreaks, and health promotion. This journal welcomes submissions that document the outbreak and contribute to the control of the disease.

Dear readers,

For the next few issues, we will publish a selection of letters and short papers that we are receiving on the COVID-19 epidemic. In this day and age, there are more rapid means of communication, but the printed word remains unsurpassed as long-term record of what has happened. For our journal, the teaching of public health undergraduate and postgraduate students is important, and this will be a useful resource.

The Editors
